# Different sites of actions make different responses to thiazolidinediones between mouse and rat models of fatty liver

**DOI:** 10.1038/s41598-021-04036-7

**Published:** 2022-01-10

**Authors:** Chihiro Ebihara, Megumi Aizawa-Abe, Mingming Zhao, Valentino Gumbilai, Ken Ebihara

**Affiliations:** 1 Department of Medicine and Clinical Science, Kyoto University Graduate School of Medicine, Kyoto, Japan; 2 Division of Endocrinology and Metabolism, Department of Internal Medicine, Jichi Medical University School of Medicine, 3311-1 Yakushiji, Shimotsuke-shi, Tochigi 329-0498 Japan; 3 Institute for Advancement of Clinical and Translational Science, Kyoto University Hospital, Kyoto, Japan

**Keywords:** Non-alcoholic fatty liver disease, Metabolic syndrome, Diabetes complications

## Abstract

Therapeutic approach for NAFLD is limited and there are no approved drugs. Pioglitazone (PGZ), a thiazolidinedione (TZD) that acts via peroxisome proliferator activated receptor gamma (PPAR*γ*) is the only agent that has shown consistent benefit and efficacy in clinical trials. However, the mechanism of its therapeutic effect on NAFLD remains unclear. The poor understanding may be due to problems with mouse, a species most used for animal experiments. TZDs exacerbate fatty liver in mouse models while they improve it in rat models like in human patients. Therefore, we compared the effects of TZDs including PGZ and rosiglitazone (RGZ) in *ob/ob* mice and *Lep*^*mkyo*^*/Lep*^*mkyo*^ rats, models of leptin-deficient obesity, and A-ZIP/F-1 mice and seipin knockout (SKO) rats, models of generalized lipodystrophy. *Pparg* mRNA expression was markedly upregulated in fatty livers of mouse models while it was unchanged in rat models. TZDs exacerbated fatty liver in *ob/ob* and A-ZIP/F-1 mice, improved it in *Lep*^*mkyo*^*/Lep*^*mkyo*^ rats and showed no effect in SKO rats. Gene expression analyses of *Pparg* and its target gene, Fsp27 revealed that PPAR*γ* in the adipose tissue is the exclusive therapeutic target of TZDs in rats but PPAR*γ* in the liver in addition to the adipose tissue is also a major site of actions for TZDs in mice. Although the response to TZDs in mice is the complete opposite of that in human patients, no report has pointed out the problem with TZD studies using mouse models so far. The present study might provide useful suggestions in research on TZDs.

## Introduction

Metabolic syndrome, a complex risk factor for cardiovascular disease, predicts the development of non-alcoholic fatty liver disease (NAFLD)^1,2^. NAFLD is now recognized as the hepatic component of the metabolic syndrome^3^. In addition, metabolic syndrome with its individual components is also a major risk factor for the development of nonalcoholic steatohepatitis (NASH), the most severe form of NAFLD^4^. NASH can progress to cirrhosis, hepatocellular carcinoma, and liver failure. Despite the increasing number of patients, therapeutic approaches for NAFLD and NASH are limited and there are no approved drug treatments^5^. There is an urgent need for further research in this field.

Many clinical trials have been and are being conducted for the drug treatment of NAFLD and NASH^6^. Among these drugs, pioglitazone (PGZ) is the only agent that has demonstrated consistent benefit and efficacy in clinical trials^7–11^. PGZ is a thiazolidinedione (TZD) that acts by binding to peroxisome proliferator activated receptor gamma (PPAR*γ*)^12^. PPAR*γ* is expressed mainly in adipocytes and plays a key role in lipid metabolism and glucose regulation^13^. Thus, PGZ is used as an antidiabetic agent to improve adipocyte dysfunction and insulin resistance^14^. However, the mechanism of its therapeutic effect on NAFLD still remains unclear. Since insulin resistance is closely related to the fat accumulation in the liver, one of the mechanisms is thought to be the insulin sensitization^15,16^, but metformin did not show any effect on NAFLD so far, despite its effectiveness in improving insulin resistance ^17,18^. Thus, the insulin sensitization by itself does not explain the therapeutic effect of PGZ on NAFLD.

The poor understanding of the mechanism by which PGZ improves NAFLD is mainly due to the species most commonly used in animal experiments for medical research today. In contrast to human patients, TZDs including PGZ exacerbate fatty liver in many mouse models^19–21^. A series of studies with mouse models revealed tissue-specific effects of PPAR*γ*, the canonical target of TZDs, on NAFLD. PPAR*γ* overexpression in the liver induces hepatic steatosis^22^, whereas liver-specific PPAR*γ* disruption prevents fat accumulation in the liver of mouse models of NAFLD^23–25^. On the other hand, muscle-specific or adipocyte-specific disruption promotes hepatic steatosis^26,27^. Thus, all these tissues, including the liver, the adipose tissue, and the skeletal muscle, may be responsible for the different responses to TZDs between mouse and human.

Conversely, it has been reported that TZDs improve fatty liver in rat models as well as in human patients^28–30^. However, due to the small number of genetically engineered rats, the tissue-specific effects of PPAR*γ* on NAFLD have not really been investigated in rat models. Under these circumstances, we have generated disease-model rats including *Lep*^*mkyo*^*/Lep*^*mkyo*^ and seipin knockout (SKO) rats^31–33^. *Lep*^*mkyo*^*/Lep*^*mkyo*^ rats have a nonsense mutation in leptin gene and exhibit hyperphagia and obese phenotypes including severe fatty liver^31^. Seipin is a protein encoded by *BSCL2* gene, whose mutation causes the most severe variety of congenital generalized lipodystrophy (CGL) in human subjects^34^. CGL is a disease characterized by a near total lack of adipose tissue from birth^35^. CGL patients develop severe insulin resistance, hypertriglyceridemia and fatty liver^36^. SKO rats have a nonsense mutation in *BSCL2* gene and exhibit phenotypes of CGL including severe insulin resistance, hypertriglyceridemia and fatty liver^32^. As mouse counterparts of *Lep*^*mkyo*^*/Lep*^*mkyo*^ and SKO rats, *ob/ob* and A-ZIP/F-1 mice are available. *ob/ob* mice also have a nonsense mutation in leptin gene and exhibit obese phenotypes including severe fatty liver^37^. A-ZIP/F-1 mice express adipose-specifically a dominant-negative protein that prevents the DNA binding of B-ZIP transcription factors including C/EBP and Jun families and exhibit phenotypes of CGL including severe fatty liver^38^. In both *ob/ob* and A-ZIP/F-1 mice, TZDs were shown to aggravate fatty liver^20,21^. The presence of mouse and rat models of obesity or lipodystrophy prompted us to explore the mechanism of the different response to TZDs by species and tissue-specific effects of PPAR*γ* on NAFLD.

In this study, *ob/ob* or A-ZIP/F-1 mice, and *Lep*^*mkyo*^*/Lep*^*mkyo*^ or SKO rats were treated with PGZ or rosiglitazone (RGZ). RGZ is another TZD available in a clinical setting, although its use is limited due to the risk of cardiovascular events^11^. After 4 weeks of each treatment, we examined fat accumulation in the liver, white adipose tissue and skeletal muscle. These tissues have been reported to have a role in PPAR*γ* action^22–27^. We also examined *Pparg* mRNA expression and PPAR*γ* target gene, *Fsp27* and *Cd36* mRNA expressions as indices of PPAR*γ* activity in these tissues. The present study revealed not only the cause of the different response to TZDs between mouse and rat, but also the mechanism by which TZDs attenuate fatty liver in rat. These findings might be helpful for a better understanding of the action mechanism of TZDs in human and the optimal treatment of TZDs for NAFLD.

## Results

### *Pparg* mRNA expressions in fatty livers from mouse and rat models of leptin deficient obesity and generalized lipodystrophy

**Figure 1:**
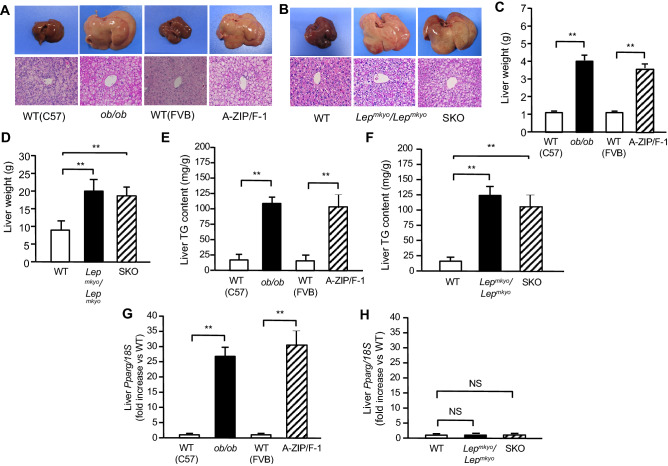
Liver phenotype and liver *Pparg* mRNA expression in mouse and rat models of leptin deficient obesity and generalized lipodystrophy. (**A**,**B**) Macroscopic (top) and histological images (bottom) of the liver, (**C**,**D**) liver weight, (**E**,**F**) liver triglyceride content and (**G**,**H**) liver *Pparg* mRNA expression in 16 weeks old male C57B/6J WT, *ob/ob*, FVB/N WT and A-ZIP/F-1 mice (**A**,**C**,**E**,**G**) and F344 WT, *Lep*^*mkyo*^*/Lep*^*mkyo*^ and SKO rats (**B**,**D**,**F**,**H**). For histological examination, hematoxylin and eosin staining was used. Original magnification of × 200 is shown. *Pparg* mRNA expression levels were normalized by 18S. Values are means ± SEM (*n* = 10 per group). *P < 0.05, **P < 0.01, NS, not significant (one-way ANOVA followed by Tukey’s test).

To explore the mechanism by which TZDs exert different effects on fatty liver between mouse and rat, we examined *Pparg* mRNA expressions in fatty liver models of mouse and rat. Namely, we used *ob/ob* mice and *Lep*^*mkyo*^*/Lep*^*mkyo*^ rats, which are leptin deficient obese models, and A-ZIP/F-1 mice and seipin KO (SKO) rats, which are models of generalized lipodystrophy. All these four models showed severe fatty liver in a similar manner when compared with each WT control (Fig. 1). Macroscopically, livers were remarkably enlarged and were lighter in color (Fig. 1A ,B). Histological examination showed large number of lipid droplets of various sizes (Fig. 1A,B). Consistent with these results, liver weight and liver TG content were both remarkably increased in all these four models regardless of species (Fig. 1C–F). However, while hepatic *Pparg* mRNA expression was upregulated in both *ob/ob* and A-ZIP/F-1 mice when compared with each WT control (Fig. 1G), it was kept at low levels in *Lep*^*mkyo*^*/Lep*^*mkyo*^ and SKO rats as with the level of their WT control (Fig. 1H). These results clearly demonstrate that *Pparg* mRNA expression is differently regulated in fatty livers between mice and rats.

To confirm these differences in the regulation of PPARγ expression at protein level, we examined PPARγ protein levels in the liver separately in cytoplasmic and nuclear fractions using western blot analysis. PPARγ protein levels were remarkably increased in both cytoplasmic and nuclear fractions in *ob/ob* and A-ZIP/F-1 mice when compared to each WT control (Supplemental Fig. 1A–C). Interestingly, the ratio of nuclear to cytoplasmic levels of PPARγ protein was also markedly increased in *ob/ob* and A-ZIP/F-1 mice (Supplemental Fig. 1D). In contrast, PPARγ protein expression was detected in neither cytoplasmic nor nuclear fractions in not only WT rats but also *Lep*^*mkyo*^*/Lep*^*mkyo*^ and SKO rats (Supplemental Fig. 1E–G). These results confirmed that PPARγ protein expression was also differently regulated in fatty livers between mice and rats.

To investigate the mechanism underlying this difference of PPAR*γ* expression, we compared basal *Pparg* mRNA expression in WT mice and rats using mean Ct values of real-time PCR. Although they were reference data as different PCR primer sets were used in mice and rats, while mean Ct values in WAT showed no significant difference between mice and rats, mean Ct value in the liver in rats was over 15 cycles more than that in mice (Supplemental Fig. 2A,B). These results indicate that *Pparg* mRNA expression in the the liver is already remarkably different between mice and rats at the basal level.

**Figure 2:**
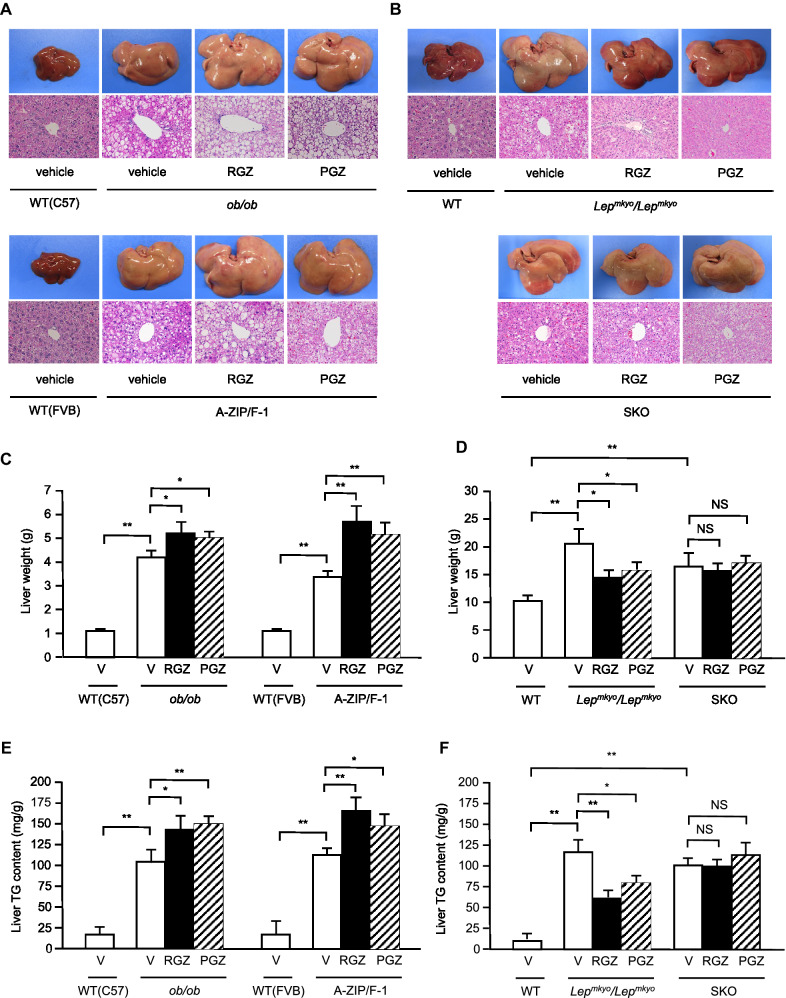
Effect of TZD treatment on fatty liver in mouse and rat models of leptin deficient obesity and generalized lipodystrophy. (**A**,**B**) Macroscopic (top) and histological images (bottom) of the liver, (**C**,**D**) liver weight and (**E**,**F**) liver triglyceride content in C57B/6J WT, *ob/ob*, FVB/N WT and A-ZIP/F-1 mice (**A**,**C**,**E**) and F344 WT, *Lep*^*mkyo*^*/Lep*^*mkyo*^ and SKO rats (**B**,**D**,**F**) treated with vehicle, RGZ or PGZ. For histological examination, hematoxylin and eosin staining was used. Original magnification of × 200 is shown. For liver weight and liver triglyceride content, values are means ± SEM (*n* = 10 per group). *P < 0.05, **P < 0.01, NS, not significant (one-way ANOVA followed by Tukey’s test).

### Effects of TZD treatment on fatty liver in mouse and rat models of leptin deficient obesity and generalized lipodystrophy

We treated *ob/ob* and A-ZIP/F-1 mice and *Lep*^*mkyo*^*/Lep*^*mkyo*^ and SKO rats with RGZ or PGZ for 4 weeks. Both RGZ and PGZ showed no effect on food intake in any of the four animal models when compared to the respective vehicle-treated controls (Supplemental Fig. 3A,B). At this time, RGZ and PGZ similarly increased the size of the liver macroscopically in *ob/ob* and A-ZIP/F-1 mice (Fig. 2A). Histological examination also showed that RGZ and PGZ increased the number and size of lipid droplets in the livers of *ob/ob* and A-ZIP/F-1 mice (Fig. 2A). Consistent with these results, both RGZ and PGZ significantly increased liver weight and liver TG content in *ob/ob* and A-ZIP/F-1 mice (Fig. 2B,C). In contrast to the mouse models, RGZ and PGZ similarly decreased the size of the liver macroscopically and decreased the number and the size of lipid droplets in the liver histologically in *Lep*^*mkyo*^*/Lep*^*mkyo*^ rats (Fig. 2D). Liver weight and liver TG content were also significantly decreased by RGZ and PGZ in *Lep*^*mkyo*^*/Lep*^*mkyo*^ rats (Fig. 2E,F). On the other hand, neither RGZ nor PGZ showed any effect on the size of the liver and lipid droplet accumulation in the liver in SKO rats (Fig. 2D). Consistent with these results, liver weight and liver TG content were also unchanged by both RGZ and PGZ in SKO rats (Fig. 2E,F). The different effects of TZDs on fatty liver in mice and rats could be explained by the different regulation of *Pparg* mRNA expression in the liver, but the different effects of TZDs in *Lep*^*mkyo*^*/Lep*^*mkyo*^ and SKO rats could be due to causes other than hepatic PPAR*γ*.

**Figure 3:**
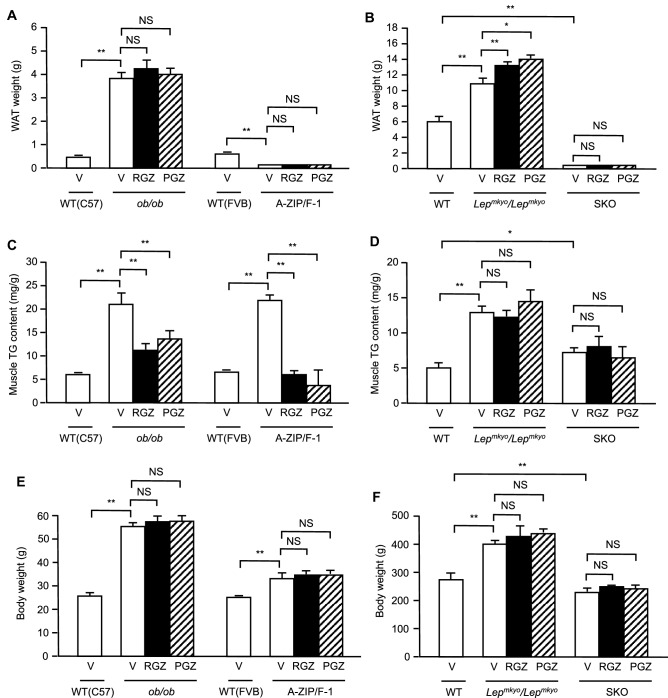
Effect of TZD treatment on WAT and skeletal muscle in mouse and rat models of leptin deficient obesity and generalized lipodystrophy. (**A**,**B**) epididymal WAT weight and (**C**,**D**) gastrocnemius muscle triglyceride content in C57B/6J WT, *ob/ob*, FVB/N WT and A-ZIP/F-1 mice (**A**,**C**) and F344 WT, *Lep*^*mkyo*^*/Lep*^*mkyo*^ and SKO rats (**B**,**D**) treated with vehicle, RGZ or PGZ. Values are means ± SEM (*n* = 10 per group). *P < 0.05, **P < 0.01, NS, not significant (one-way ANOVA followed by Tukey’s test). ND, not determined.

### Effects of TZD treatment on WAT weight, muscle TG content and body weight in mouse and rat models of leptin deficient obesity and generalized lipodystrophy

We next measured epididymal WAT weight after 4 weeks of RGZ or PGZ treatment to investigate the effect of TZDs on TG accumulation in WAT which is the major expression site of PPAR*γ*. Without TZD treatment, epididymal WAT weight was markedly increased in *ob/ob* mice and *Lep*^*mkyo*^*/Lep*^*mkyo*^ rats when compared with each WT control (Fig. 3A,B). Both RGZ and PGZ further increased WAT weight in *Lep*^*mkyo*^*/Lep*^*mkyo*^ rats but not in *ob/ob* mice (Fig. 3A,B). In A-ZIP/F-1 mice and SKO rats, models of generalized lipodystrophy, the amount of remaining WAT was marginal and inadequate for analysis and neither RGZ nor PGZ had any significant effect (Fig. 3A,B). We also measured TG content in gastrocnemius muscle to investigate the effects of TZDs on TG accumulation in the skeletal muscle. Without TZD treatment, muscle TG content was apparently increased in all four models when compared to the respective vehicle-treated WT controls (Fig. 3C,D). Both RGZ and PGZ significantly decreased muscle TG content in *ob/ob* and A-ZIP/F-1 mice, but not in *Lep*^*mkyo*^*/Lep*^*mkyo*^ and SKO rats (Fig. 3C,D). While the effects of TZDs on liver weight, WAT weight, and muscle TG content were varied by animal models, body weight was largely unchanged in both RGZ and PGZ in all four models (Fig. 3E,F).

### Effects of TZD treatment on glucose metabolism in mouse and rat models of leptin deficient obesity and generalized lipodystrophy

**Figure 4:**
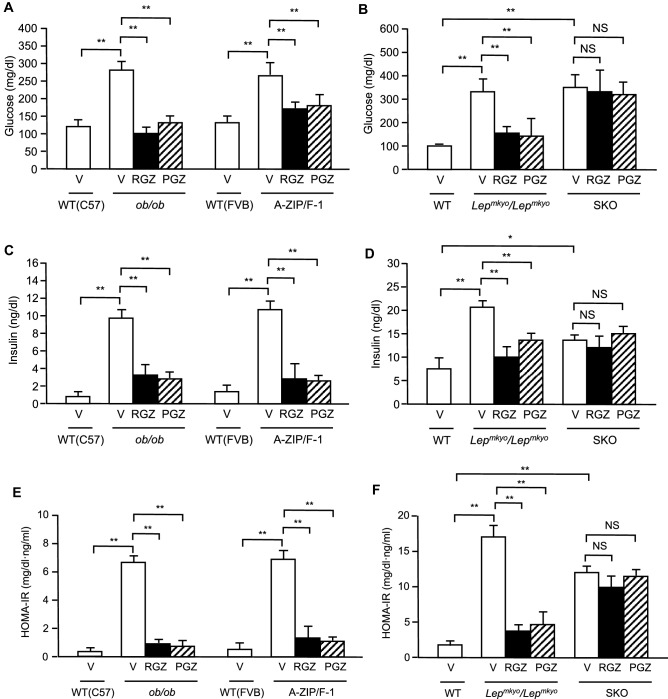
Effect of TZD treatment on glucose metabolism in mouse and rat models of leptin deficient obesity and generalized lipodystrophy. (**A**,**B**) Plasma glucose and (**C**,**D**) insulin concentrations in C57B/6J WT, *ob/ob*, FVB/N WT and A-ZIP/F-1 mice (**A**,**C**) and F344 WT, *Lep*^*mkyo*^*/Lep*^*mkyo*^ and SKO rats (**B**,**D**) treated with vehicle, RGZ or PGZ. Values are means ± SEM (*n* = 10 per group). *P < 0.05, **P < 0.01, NS, not significant (one-way ANOVA followed by Tukey’s test).

To evaluate the effects of TZDs on glucose metabolism, we measured fasting plasma glucose and insulin concentrations after 4 weeks of RGZ or PGZ treatment. Without TZD treatment, fasting plasma glucose and insulin concentrations were apparently increased in all four models compared to each WT control (Fig. 4A–D). Therefore, HOMA-IR, an indicator of insulin resistance, was markedly increased in all these four models (Fig. 4E,F). However, RGZ and PGZ effectively decreased fasting plasma glucose and insulin concentrations and HOMA-IR in three of four models, except SKO rats (Fig. 4A–F). In SKO rats, fasting plasma glucose and insulin concentrations and HOMA-IR were almost unchanged by both RGZ and PGZ.

### Effects of TZD treatment on lipid metabolism in mouse and rat models of leptin deficient obesity and generalized lipodystrophy

**Figure 5:**
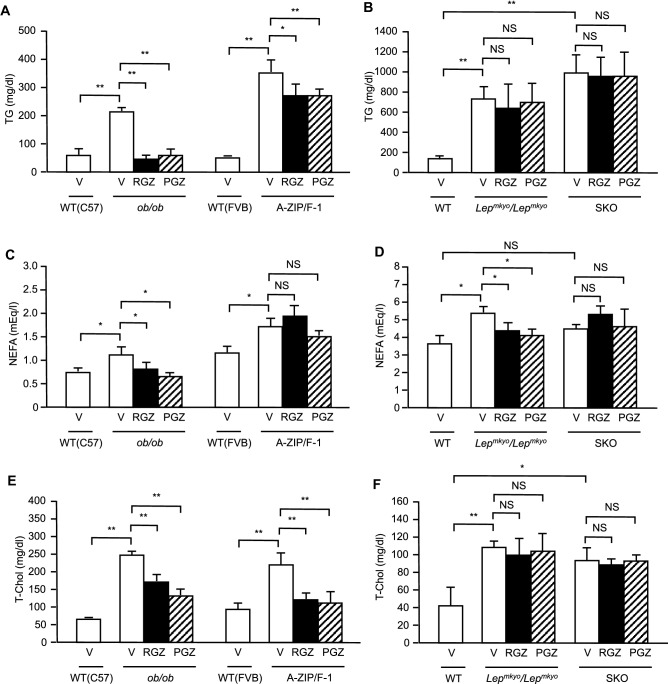
Effect of TZD treatment on lipid metabolism in mouse and rat models of leptin deficient obesity and generalized lipodystrophy. (**A**,**B**) Plasma triglyceride, (**C**,**D**) NEFA and (**E**,**F**) total cholesterol concentrations in C57B/6J WT, *ob/ob*, FVB/N WT and A-ZIP/F-1 mice (**A**,**C**,**E**) and F344 WT, *Lep*^*mkyo*^*/Lep*^*mkyo*^ and SKO rats (**B**,**D**,**F**) treated with vehicle, RGZ or PGZ. Values are means ± SEM (*n* = 10 per group). *P < 0.05, **P < 0.01, NS, not significant (one-way ANOVA followed by Tukey’s test).

The effects of TZDs on lipid metabolism were also examined in our mouse and rat models. Without TZD treatment, fasting plasma TG, NEFA, and total cholesterol concentrations were apparently increased in all four models compared to WT controls (Fig. 5A–F). With TZDs treatment, fasting plasma TG concentration was massively decreased in *ob/ob* mice and moderately decreased in A-ZIP/F-1 mice (Fig. 5A,B). In contrast, TZDs had no effect on fasting plasma TG concentrations in rat models, *Lep*^*mkyo*^*/Lep*^*mkyo*^ and SKO rats (Fig. 5A,B). In obese models, *ob/ob* mice and *Lep*^*mkyo*^*/Lep*^*mkyo*^ rats, TZDs resulted a slight but significant decrease in fasting plasma NEFA concentrations (Fig. 5C,D). On the other hand, TZDs had no effect on fasting plasma NEFA concentrations in models of generalized lipodystrophy, A-ZIP/F-1 mice and SKO rats (Fig. 5C,D). Plasma total cholesterol concentrations were substantially decreased in mouse models by TZDs while TZDs had no effect on plasma total cholesterol concentrations in rat models (Fig. 5E,F).

### Effects of TZD treatment on *Pparg* and its target genes mRNA expressions in the liver, WAT and skeletal muscle

To investigate the mechanism underlying the different effect of TZDs among animal models, we examined mRNA expressions of *Pparg* and its target genes, *Fsp27* and *Cd36* in epididymal WAT, liver and gastrocnemius muscle of our four animal models after 4 weeks of RGZ or PGZ treatment.

**Figure 6:**
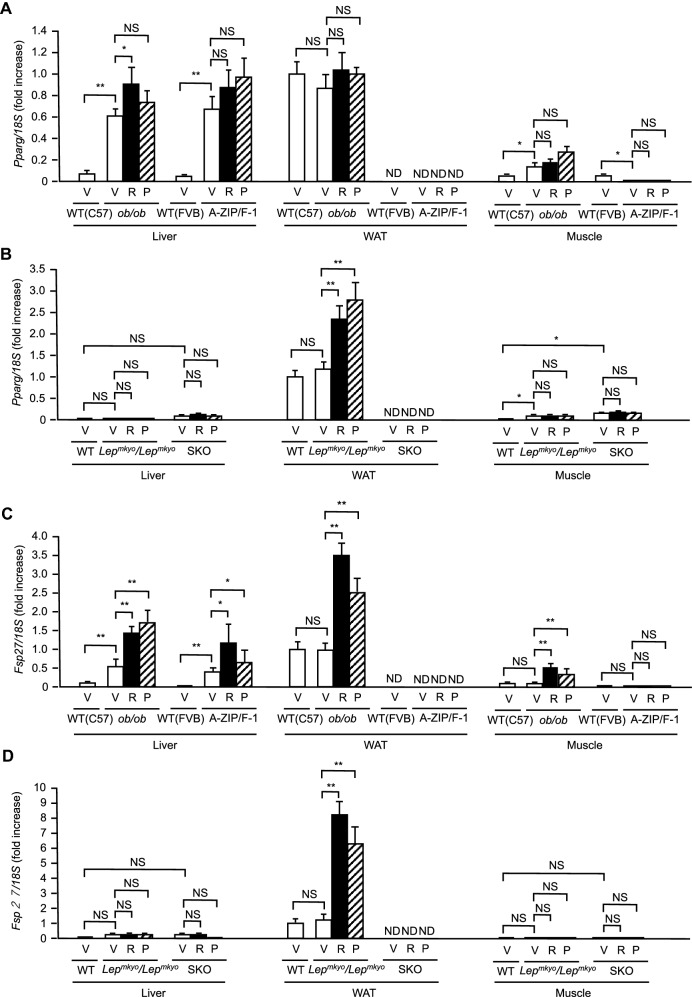
Effect of TZD treatment on *Pparg* and *Fsp27* mRNA expressions in the liver, WAT and skeletal muscle in mouse and rat models of leptin deficient obesity and generalized lipodystrophy. (**A**,**B**) *Pparg* mRNA expressions in the liver, WAT and skeletal muscle in C57B/6J WT, *ob/ob*, FVB/N WT and A-ZIP/F-1 mice (**A**) and F344 WT, *Lep*^*mkyo*^*/Lep*^*mkyo*^ and SKO rats (**B**) treated with vehicle, RGZ or PGZ. (**C**,**D**) *Fsp27* mRNA expressions in the liver, WAT and skeletal muscle in C57B/6 J WT, *ob/ob*, FVB/N WT and A-ZIP/F-1 mice (**C**) and F344 WT, *Lep*^*mkyo*^*/Lep*^*mkyo*^ and SKO rats (**D**) treated with vehicle, RGZ or PGZ. mRNA expression levels were normalized by 18S. Values are means ± SEM (*n* = 10 per group). ND, not determined. *P < 0.05, **P < 0.01, NS, not significant (one-way ANOVA followed by Tukey’s test).

Consistent with the baseline results (Fig. 1G,H), hepatic *Pparg* mRNA expression was very low in vehicle-treated WT controls irrespective of mice or rats, but markedly increased in vehicle-treated mouse models, *ob/ob* and A-ZIP/F-1 mice while it was kept at low levels in vehicle-treated rat models, *Lep*^*mkyo*^*/Lep*^*mkyo*^ and SKO rats (Fig. 6A,B). Hepatic *Fsp27* and *Cd36* mRNA expressions were also very low in vehicle-treated WT controls irrespective of mice or rats, but were increased in vehicle-treated mouse models while it was kept at low levels in vehicle-treated rat models (Fig. 6C,D, Supplemental Fig. 4A,B). In mouse models, although both RGZ and PGZ showed subtle effects on hepatic *Pparg* mRNA expression itself, its target *Fsp27* and *Cd36* mRNA expressions were significantly further increased by both RGZ and PGZ (Fig. 6A,C, Supplemental Fig. 4A). On the other hand, neither RGZ nor PGZ increased the hepatic *Pparg* mRNA expression as well as the hepatic *Fsp27* and *Cd36* mRNA expressions in rat models (Fig. 6B,D, Supplemental Fig. 4B).

In WAT, substantial expression of *Pparg* mRNA was detected in vehicle-treated WT controls irrespective of mouse or rat, and was unchanged in both vehicle-treated mouse and rat obese models (Fig. 6A,B). Consistent with these results, substantial expressions of *Fsp27* and *Cd36* mRNA were also detected in WAT in both vehicle-treated WT mouse and rat controls, and was almost unchanged in both vehicle-treated mouse and rat obese models (Fig. 6C,D, Supplemental Fig. 4A,B). Both RGZ and PGZ showed no significant effect on WAT *Pparg* mRNA expression in *ob/ob* mice, but were significantly increased in *Lep*^*mkyo*^*/Lep*^*mkyo*^ rats (Fig. 6A,B). RGZ and PGZ showed different effects on WAT *Pparg* mRNA expression in *ob/ob* mice and *Lep*^*mkyo*^*/Lep*^*mkyo*^ rats, but both effectively increased PPAR*γ* target genes, *Fsp27* and *Cd36* mRNA expressions in WAT in both mouse and rat obese models, indicating that TZDs enhanced WAT PPAR*γ* activity similarly in mice and rats (Fig. 6C,D, Supplemental Fig. 4A,B). Since the amount of remaining WAT was marginal and inadequate for analysis, we did not examine WAT mRNA expressions in A-ZIP/F-1 mice and SKO rats, models of generalized lipodystrophy (Fig. 6A–D, Supplemental Fig. 4A,B).

In the skeletal muscle, *Pparg* mRNA expression was basically at low levels in both mice and rats (Fig. 6A,B). Although muscle *Pparg* mRNA expression was increased marginally in *ob/ob* mice and *Lep*^*mkyo*^*/Lep*^*mkyo*^ rats, TZDs showed no significant effect on it (Fig. 6A,B). In accordance with *Pparg* mRNA expressions, muscle *Fsp27* and *Cd36* mRNA expression was also basically at low levels in both mice and rats although TZDs increased it slightly only in *ob/ob* mice (Fig. 6C,D, Supplemental Fig. 4A,B).

## Discussion

There have been no reports directly comparing hepatic *Pparg* mRNA expression between mouse and rat so far. The present study clearly demonstrated the different regulation of hepatic *Pparg* mRNA expression between mouse and rat. PPAR*γ* mRNA expression was markedly upregulated in fatty liver of mouse models compared to WT controls, while it was unchanged in rat models (Fig. 1G,H). We also confirmed that PPARγ expression is differently regulated at protein level (Supplemental Fig. 1). In this study, we used leptin-deficient obese and generalized lipodystrophy mice and rats, respectively, as fatty liver models. We confirmed the degree of fat accumulation in the liver was almost the same in all these four models irrespective of species (Fig. 1E,F). Thus, the difference in hepatic *Pparg* mRNA expression between mouse and rat is not due to the differences in fat accumulation in the liver. Although the molecular mechanism underlying the different regulation of hepatic *Pparg* mRNA expression between the two species remains unclear, this difference could explain the different hepatic responses of PPAR*γ* agonists, TZDs between mouse and rat.

As previously reported^20,21^, TZDs exacerbated fatty liver in *ob/ob* and A-ZIP/F-1 mice with upregulated hepatic *Pparg* expression (Fig. 2A,C,E). In contrast, TZDs improved fatty liver in leptin-deficient *Lep*^*mkyo*^*/Lep*^*mkyo*^ rats without upregulation of hepatic *Pparg* expression (Fig. 2B,D,F), as previously reported in leptin receptor-deficient Zucker fatty rats^29^. Surprisingly, TZDs showed completely no effect on fatty liver in SKO rats which also had no upregulation of hepatic *Pparg* expression (Fig. 2B,D,F). Since SKO rats have a near total lack of white adipose tissue^32^, this result indicate that adipose tissue is necessary for the therapeutic effect of TZDs on fatty liver in rats. Furthermore, the results also indicate that PPAR*γ* in other tissues, such as liver and skeletal muscle, are not physiologically significant for the therapeutic effect of TZDs in rats. Therefore, it can be said that TZDs improved fatty liver in *Lep*^*mkyo*^*/Lep*^*mkyo*^ rats mainly through adipose tissue. In both *ob/ob* and A-ZIP/F-1 mice, TZDs markedly decreased muscle TG content while significantly increased liver TG content (Fig. 2E and 3C). Since PPAR*γ* is a transcriptional factor that induces the expression of lipogenic genes, these results indicate that PPAR*γ* in skeletal muscle is not the major site of actions for TZDs even in mice. Taken together, PPAR*γ* in the adipose tissue is the exclusive therapeutic target of TZDs in rats, while PPAR*γ* in the liver in addition to that in the adipose tissue is also a major site of actions for TZDs in mice.

To verify the significance of PPAR*γ* as a therapeutic target of TZDs in each tissue, we examined the expressions of *Pparg* and its target genes, *Fsp27* and *Cd36,* in the liver, skeletal muscle and adipose tissue. In mice, high expression of *Pparg* was detected, and TZDs significantly increased the expressions of *Fsp27* and *Cd36* in both liver and adipose tissue (Fig. 6A,C, Supplemental Fig. 4A). In *ob/ob* mice but not in A-ZIP/F-1 mice, although the expression of *Pparg* was slightly but significantly upregulated and TZDs increased the expressions of *Fsp27* and *Cd36* in skeletal muscle, the increases of these expressions were very small when compared with those in the liver and adipose tissue (Fig. 6A,C, Supplemental Fig. 4A). On the other hand, the expression of *Pparg* was negligible and TZDs had no effect on the expressions of *Fsp27* and *Cd36* in both liver and skeletal muscle in rat models (Fig. 6B,D, Supplemental Fig. 4B). The amount of *Pparg* expression and the transcriptional activity of PPAR*γ* measured by *Fsp27* and *Cd36* expressions indicate that PPAR*γ* in adipose tissue is the exclusive therapeutic target of TZDs in rats and that PPAR*γ* in the liver in addition to adipose tissue is also the major site of actions for TZDs in mice. Moreover, the significance of PPAR*γ* in skeletal muscle is minimal, if any, in both mice and rats.

**Figure 7:**
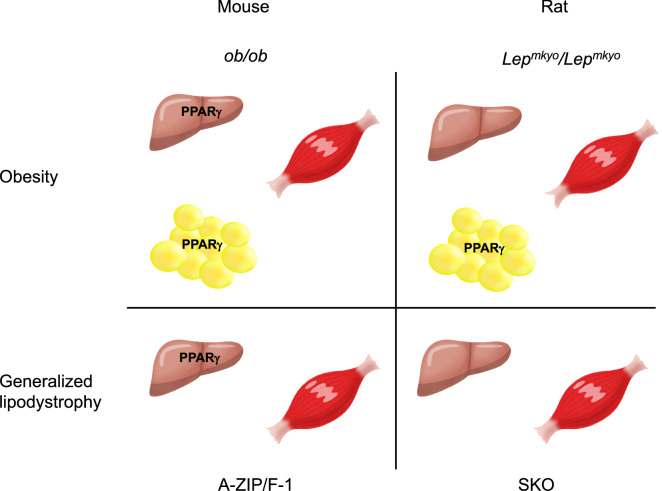
Expression sites of physiologically functional PPARg in mouse and rat models of fatty liver. The therapeutic target tissues of TZDs are adipose tissue and liver in *ob/ob* mice, liver only in A-ZIP/F-1 mice, adipose tissue only in *Lep*^*mkyo*^*/Lep*^*mkyo*^ rats, and no tissue in SKO rats.

As mentioned above, the therapeutic target tissues of TZDs are adipose tissue and liver in *ob/ob* mice, liver only in A-ZIP/F-1 mice, adipose tissue only in *Lep*^*mkyo*^*/Lep*^*mkyo*^ rats, and no tissue in SKO rats (Fig. 7). In the present study, the insulin sensitizing effect of TZDs was observed in A-ZIP/F-1 fatless mice other than *ob/ob* mice and *Lep*^*mkyo*^*/Lep*^*mkyo*^ rats (Fig. 4E,F). In the previous report, RGZ showed no insulin sensitizing effect in A-ZIP/F-1 mice^21^. However, the subsequent study demonstrated that RGZ treatment decrease tissue TG content and improves insulin sensitivity in skeletal muscle while it aggravates fatty liver and insulin resistance in liver in A-ZIP/F-1 mice^39^. This means that the degree of insulin sensitizing effect of TZDs in A-ZIP mice is determined by the balance between the negative effect in the liver and the positive effect in the skeletal muscle. This balance might be changed by the dose or treatment duration of TZDs. On the other hand, TZDs had no insulin sensitizing effect in SKO rats, which have no target tissue for TZDs.

As to the lipid metabolism, TZDs also showed no effect in SKO rats, which have no target tissue for TZDs. In *Lep*^*mkyo*^*/Lep*^*mkyo*^ rats which have only adipose tissue as a target for TZDs, plasma NEFA concentration was slightly decreased by TZDs. In A-ZIP/F-1 mice, which have only the liver as a target for TZDs, plasma TG and total cholesterol concentrations were significantly decreased by TZDs. In *ob/ob* mice, which have both adipose tissue and liver as a target for TZDs, plasma TG, NEFA and total cholesterol concentrations were all significantly decreased by TZDs. These results indicate that TZDs decrease plasma TG and total cholesterol concentrations through PPAR*γ* in the liver and mildly decrease plasma NEFA concentrations through PPAR*γ* in adipose tissue. Conversely, TZDs decrease neither plasma TG nor total cholesterol concentrations without functional PAR*γ* in the liver and do not decrease plasma NEFA concentrations without adipose tissues.

It has been shown that TZDs improve fatty liver in human patients as well as in rat models^7–11^. On the other hand, it has been reported that *Pparg* expression in the liver was upregulated in obese patients with NAFLD^40^. *Pparg* expressions in the liver were comparable to those in the adipose tissue in mouse models but not in rat models of fatty liver (Fig. 6A,B). However, there was no data on the comparison of *Pparg* expression between liver and adipose tissue of human patients. Thus, the pathophysiological significance of PPAR*γ* in the liver of human patients is still unclear. It has also been reported that in obese NAFLD patients, *Pparg* expression in the liver showed a positive association with mRNA expression of sterol regulatory element-binding protein (SREBP)-1c, one of most important genes for lipogenesis in the liver^40^. If PPAR*γ* plays an essential role in the induction of SREBP-1c in the liver, TZD treatment would exacerbate NAFLD in humans. However, the fact is completely opposite to what expected^7–11^. Furthermore, there is no clear evidence on the efficacy of TZDs in patients with generalized lipodystrophy so far. Although we also have some experiences in using PGZ in patients with generalized lipodystrophy, its effectiveness was very limited^41,42^. These facts indicate that PPAR*γ* in adipose tissue is the exclusive therapeutic target of TZDs in human patients as well as in rats, and that the significance of PPAR*γ* in the liver and skeletal muscle is marginal, if any, even in human subjects.

In conclusion, the present study clearly demonstrated the different regulation of hepatic *Pparg* mRNA expression between mouse and rat, TZDs on fatty liver between mouse and rat. Although the response to TZDs in mice is the exact opposite of that in human patients, there have been no reports pointing out problems with TZD researches using mouse models so far. We should be careful to interpret data on effects of TZDs obtained from mouse models. On the other hand, rat models, which show therapeutic effects of TZDs similar to those in human patients, can provide numerous useful suggestions on the TZD research.

## Methods

### Animals

*ob/ob* mice on C57BL/6J background were purchased from Japan SLC, Inc. (Hamamatsu, Japan). A-ZIP/F-1 mice on FVB/N background were provided from Diabetes Branch, National Institute of Diabetes and Digestive and Kidney Diseases^38^. *Lep*^*mkyo*^*/ Lep*^*mkyo*^ rats and SKO rats on F344/NSlc background were generated previously^31,32^. Mice and rats were maintained on a 14 h light/10 h dark cycle (lights on 7:00 AM, lights off 9:00 PM) and fed ad libitum standard pellet diet (MF; Oriental Yeast Co., Ltd., Tokyo, Japan).

The study was conducted with approval of Institutional Animal Experiment Committee at Kyoto University. All animal experiments adhered to the ARRIVE Guidelines, were carried out with the Institutional Regulation for Animal Experiments at Kyoto University.

### TZD treatments

We treated 12-week-old male *ob/ob* mice, A-ZIP/F-1 mice, *Lep*^*mkyo*^*/Lep*^*mkyo*^ rats, SKO rats and their WT littermates were treated with RGZ (Wako Pure Chemical Industries, Ltd., Osaka, Japan) or PGZ donated by Takeda Pharmaceutical Co., Ltd. (Osaka Japan) from the age of 12 weeks for 4 weeks. RGZ was dissolved in water and administered at doses of 25 mg/kg for mice and 2.5 mg/kg for rats by oral gavage (0.2 ml for mice and 0.7 ml for rats) once daily. PGZ was dissolved in 0.01% carboxymethyl cellulose (CMC) and administered at doses of 30 mg/kg for mice and 3 mg/kg for rats by oral gavage (0.2 ml for mice and 0.7 ml for rats) once daily. For vehicle-control animals, the same amount of water or 0.01% CMC was administered. At the end of 4-week treatment, livers, epididymal fats, gastrocnemius muscles and blood were sampled after 4-h fasting.

### Liver histology

Livers were sampled at the end of the experiment, fixed in 10% neutrally buffered formalin and subsequently embedded in paraffin. Histological sections of 5 µm thickness were stained with hematoxylin and eosin, and examined by light microscopy.

### Measurement of triglyceride content in the liver and gastrocnemius muscle

Livers and gastrocnemius muscles were sampled at the end of the experiment and were immediately frozen in liquid nitrogen. Lipids were extracted with isopropyl alcohol/heptane (1:1 vol./vol.). After evaporating the solvent, lipids were resuspended in 99.5% (vol./vol.) ethanol and triglyceride content was measured by an enzymatic kit (Wako Pure Chemical Industries, Ltd.).

### Biochemical assays

Blood was obtained from the inferior vena cava for mice and from tail vein for rats. Plasma glucose, triglyceride, non-esterified fatty acid (NEFA) and total cholesterol concentrations were measured by enzymatic kits (Wako Pure Chemical Industries, Ltd.). Plasma insulin concentrations were measured by an insulin-ELISA kit (Morinaga Institute of Biological Science, Inc., Yokohama Japan).

### Real-time quantitative RT-PCR

After sampling, livers, gastrocnemius muscles, and white adipose tissues were immediately frozen in liquid nitrogen and stored at – 80 °C until use for RNA isolation. RNA was prepared using Trizol (Thermo Fisher Scientific, Waltham, MA) reagent following the supplier’s protocol. The Quality and the concentrations of the extracted RNA were checked using the Nano-Drop 2000 (Thermo Fisher Scientific). Single-stranded cDNA was synthesized from 1 µg of total RNA using SuperScript III First-Strand Synthesis System for RT-PCR, according to the manufacturer's instructions (Thermo Fisher Scientific). Quantitative RT-PCR was performed with TaqMan (Applied Biosystems, Carlsbad, CA) for housekeeping rat or mouse mitochondrial subunit *18S* rRNA and rat or mouse *Pparg* and *Cd36*, and with SYBR Green (Applied Biosystems) for rat or mouse *Fsp27* by Applied Biosystems StepOnePlus™ RT-PCR System. The sequences of primers and probe used in the present study are as follows: rat 18 s forward; 5′-GCAATTATTCCCCATGAACGA-3′, rat 18 s reverse; 5′-CAAAGGGCAGGGACTTAATCAAC-3′, probe; 5′-AATTCCCAGTAAGTGCGGGTCATAAGCTTG-3′, mouse 18 s forward; 5′-CGCGCAAATTACCCACTCCCGA-3′, mouse 18 s reverse; 5′-CGGCTACCACATCCAAGGA-3′, probe; 5′-CCAATTACAGGGCCTCGAAA-3′, rat *Pparγ* forward; 5′-CCTGCGGAAGCCCTTTGGTGACT-3′, rat *Pparγ* reverse; 5′-TGACCAGGGAGTTCCTCAAAA-3′, probe; 5′-AGCAAACTCAAACTTAGGCTCCAT-3′, mouse PPAR*γ* forward; 5′-CTTCCATCACGGAGAGGTCCACAGAGC-3′, mouse *Pparγ* reverse; 5′-AGAGCATGGTGCCTTCGC-3′, probe; 5′-ATGTCAAAGGAATGCGAGTGG-3′, rat *Fsp27* forward; 5′-GTCTCTCAGCCTTCTCTACCC-3′, rat *Fsp27* reverse; 5′- CTTGCGCTGTTCTGATGGGG-3′, mouse *Fsp27* forward; 5′- CAGGCATGTGGCAGTGAGCACGG-3′, mouse *Fsp27* reverse; 5′- GTTGGCTTCTGGGAAAGGGC-3′. For rat or mouse *Cd36*, the following Assay-on-demand primer/probe sets were used: rat *Cd36*; Rn02115446_s1, mouse *Cd36*; Mm00432403_m1 (Applied Biosystems).

### Western blot analysis

Livers were homogenized and lysed in a solution containing 20 mM Tris (pH 7.5) 150 mM NaCl, 1 mM EDTA, 1 mM EGTA, 1% Triton X-100, 2.5 mM sodium pyrophosphate and 1 mM sodium orthovanadate. Subcellular fractions were obtained using Subcellular Protein Fractionation Kit for Tissues (Thermo Fisher Scientific). Samples were separated by SDS-PAGE using 4–12% BisTris gel (Bio-Rad) and transferred to PVDF membrane (Bio-Rad). Membranes were immunoblotted with each antibody. Amersham ECL prime (GE Healthcare Life Sciences, Pittsburgh, PA) and ImageQuant LAS 4000mini (GE Healthcare Life Sciences) were used for the detection and the quantification. Antibodies used in the present study are as follows: PPAR*γ* (81B8); Cell Signaling Technology, Boston, MA), β-actin (3700; Cell Signaling Technology).

### Statistical analysis

The data were analysed using SPSS software version 20.00 (SPSS Inc. Chicago, IL, USA). Data are expressed as means ± SEM. Statistical significance was assessed by one-way ANOVA followed by Tukey’s test. *P* < 0.05 was considered statistically significant.

## Supplementary Information

Supplementary Information.
