# Supplementary material for: Different sites of actions make different responses to thiazolidinediones between mouse and rat models of fatty liver
Source: Sci Rep. 2022 Jan 10;12:449. doi: 10.1038/s41598-021-04036-7 (PMC8748829; doi:10.1038/s41598-021-04036-7)

Suppl. Fig. 1

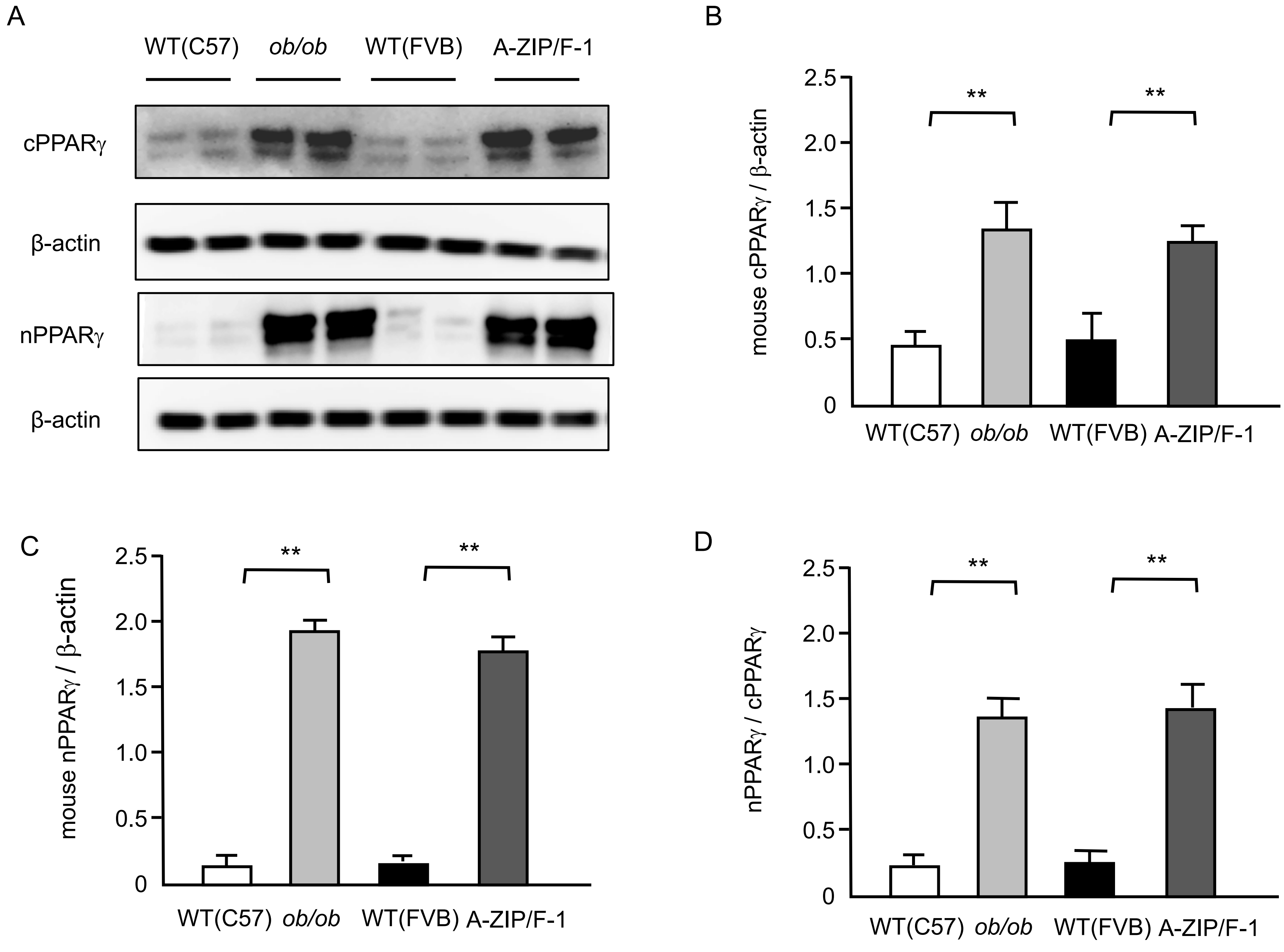

A
WT(C57)
ob/ob
WT(FVB)
A-ZIP/F-1
cPPARγ
β-actin
nPPARγ
β-actin
B
mouse cPPARγ / β-actin
2.5
2.0
1.5
1.0
0.5
0
**
**
WT(C57) ob/ob WT(FVB) A-ZIP/F-1
C
mouse nPPARγ / β-actin
2.5
2.0
1.5
1.0
0.5
0
**
**
WT(C57) ob/ob WT(FVB) A-ZIP/F-1
D
nPPARγ / cPPARγ
2.5
2.0
1.5
1.0
0.5
0
**
**
WT(C57) ob/ob WT(FVB) A-ZIP/F-1

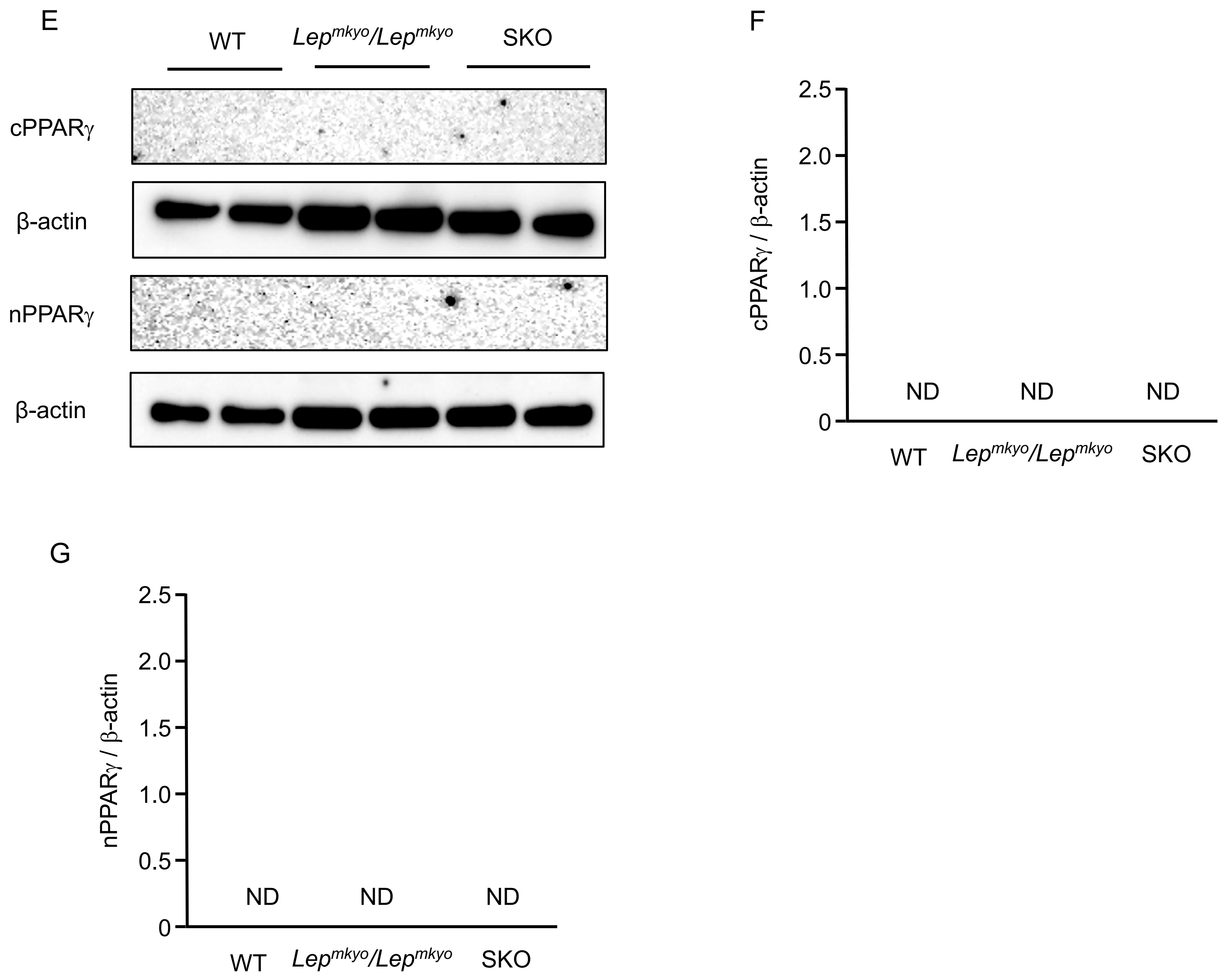
E
WT
Lepmkyo/Lepmkyo
SKO
cPPARγ
β-actin
nPPARγ
β-actin
F
cPPARγ / β-actin
2.5
2.0
1.5
1.0
0.5
0
ND
ND
ND
WT
Lepmkyo/Lepmkyo
SKO
G
nPPARγ / β-actin
2.5
2.0
1.5
1.0
0.5
0
ND
ND
ND
WT
Lepmkyo/Lepmkyo
SKO

Suppl Fig. 1. Western blot analyses for PPARγ protein levels in cytoplasmic and nuclear fractions (cPPARγ and nPPARγ) in the liver in C57B/6J WT, *ob/ob*, FVB/N WT and A-ZIP/F-1 mice (A) and F344 WT, Lep^{mkyo}/Lep^{mkyo} and SKO rats (E). (B, C, F, G) Ratio of signal intensities of cPPARγ or nPPARγ to β-actin in A and E. (D) Ratio of signal intensities of cPPARg to nPPARg in A . Values are means ± SEM (*n* = 4 per group) $^{**}P < 0.01$, ND, not detected. (one-way ANOVA followed by Tukey's test).

Suppl. Fig. 2

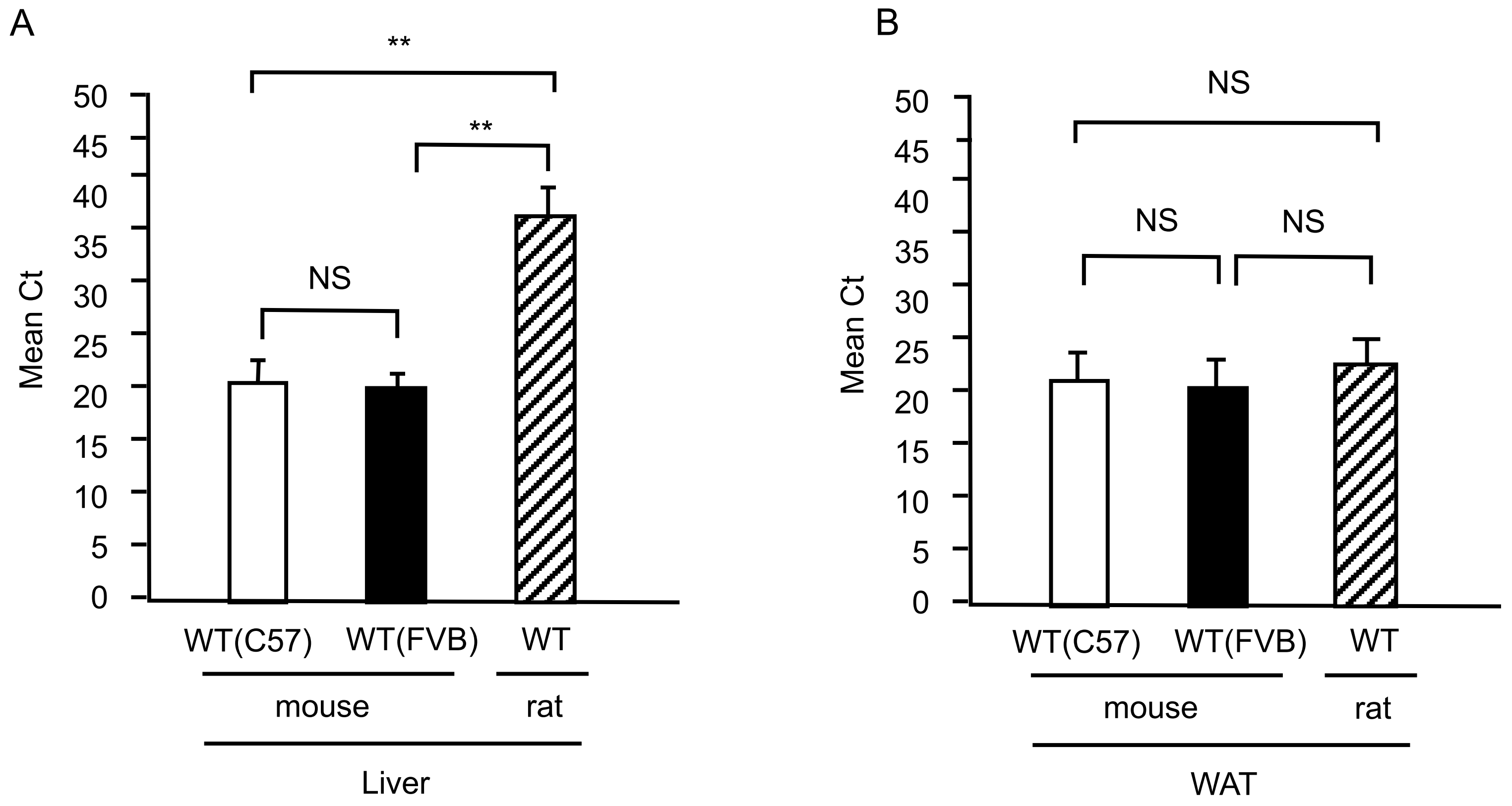

Suppl Fig. 2. Mean Ct values of *Pparg* mRNA expression by Real-time PCR in the liver (A) and WAT (B) in C57B/6J WT, FVB/N WT mice and F344 WT rats. Values are means ± SEM (n = 6 per group) $^{**}P < 0.01$, NS, not significant (one-way ANOVA followed by Tukey's test).

Suppl. Fig. 3

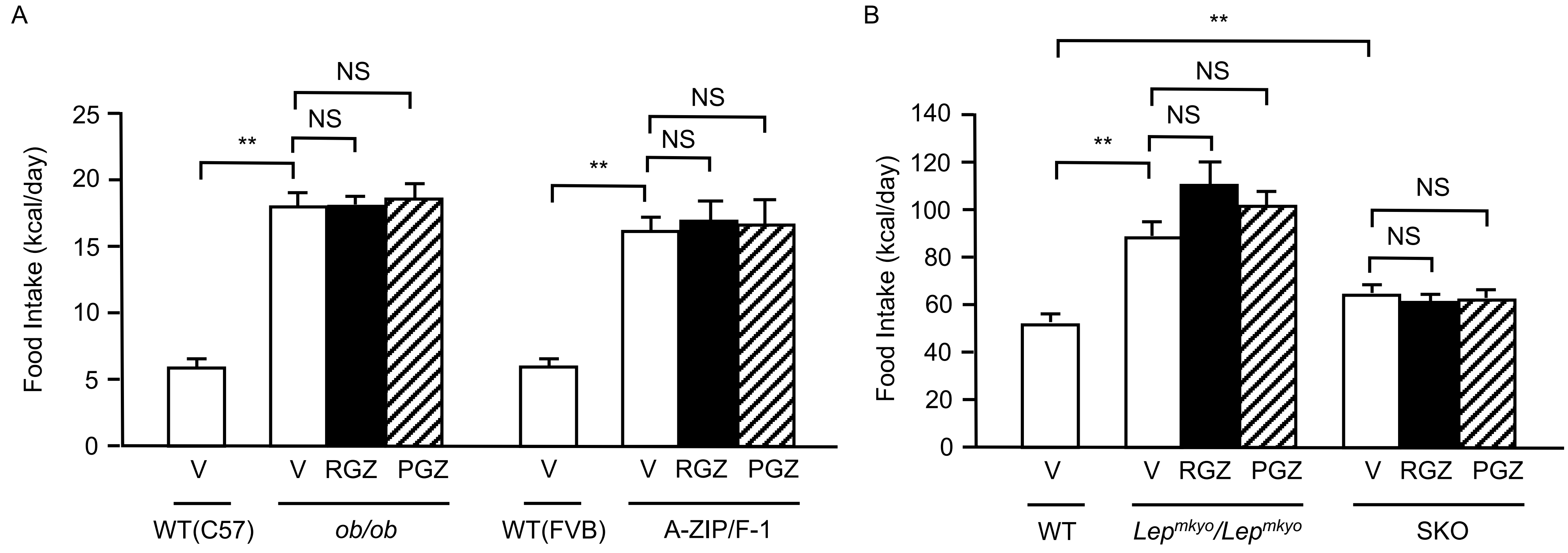

Suppl. Fig. 3. Effect of TZD treatment on food intake in mouse and rat models of leptin deficient obesity and generalized lipodystrophy. Food intake in C57B/6J WT, *ob/ob*, FVB/N WT and A-ZIP/F-1 mice (A) and F344 WT, Lep^{mkyo}/Lep^{mkyo} and SKO rats (B) treated with vehicle, RGZ or PGZ. Values are means ± SEM (n = 10 per group). **$P < 0.01$, NS, not significant (one-way ANOVA followed by Tukey's test).

Suppl. Fig. 4

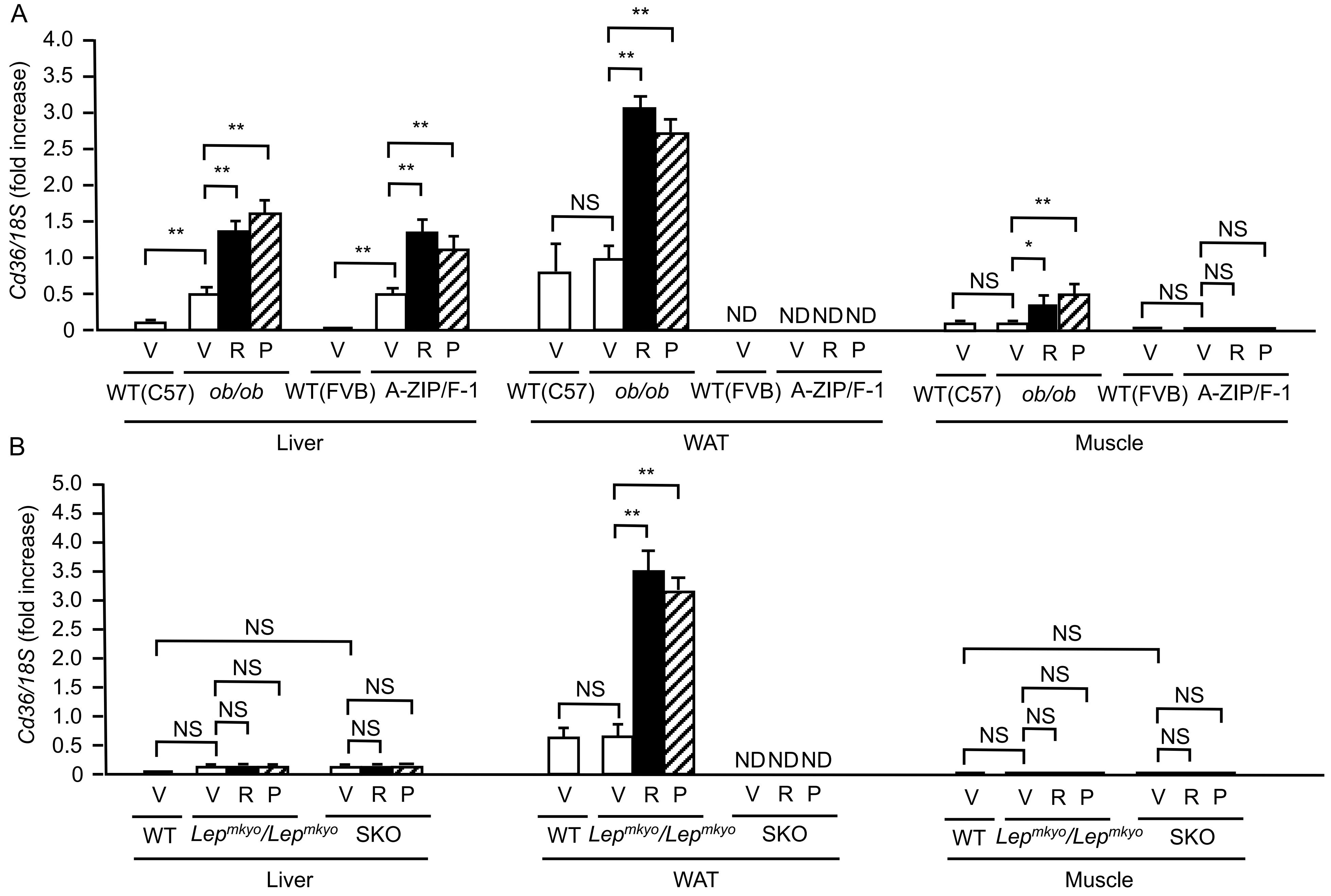
A
Cd36/18S (fold increase)
4.0
3.5
3.0
2.5
2.0
1.5
1.0
0.5
0
**
*
NS
ND
V R P
WT(C57)
ob/ob
WT(FVB)
A-ZIP/F-1
Liver
WAT
Muscle
B
5.0
4.5
WT
Lepmkyo/Lepmkyo
SKO

Suppl Fig. 4. Effect of TZD treatment on *Cd36* mRNA expressions in the liver, WAT and skeletal muscle in C57B/6J WT, *ob/ob*, FVB/N WT and A-ZIP/F-1 mice (A) and F344 WT, Lep^{mkyo}/Lep^{mkyo} and SKO rats (B) treated with vehicle, RGZ or PGZ. mRNA expression levels were normalized by 18S. Values are means ± SEM (n = 6 per group) *$P < 0.05$, **$P < 0.01$, NS, not significant (one-way ANOVA followed by Tukey's test).

Supplementary Fig. 1A (original whole gel/blot)

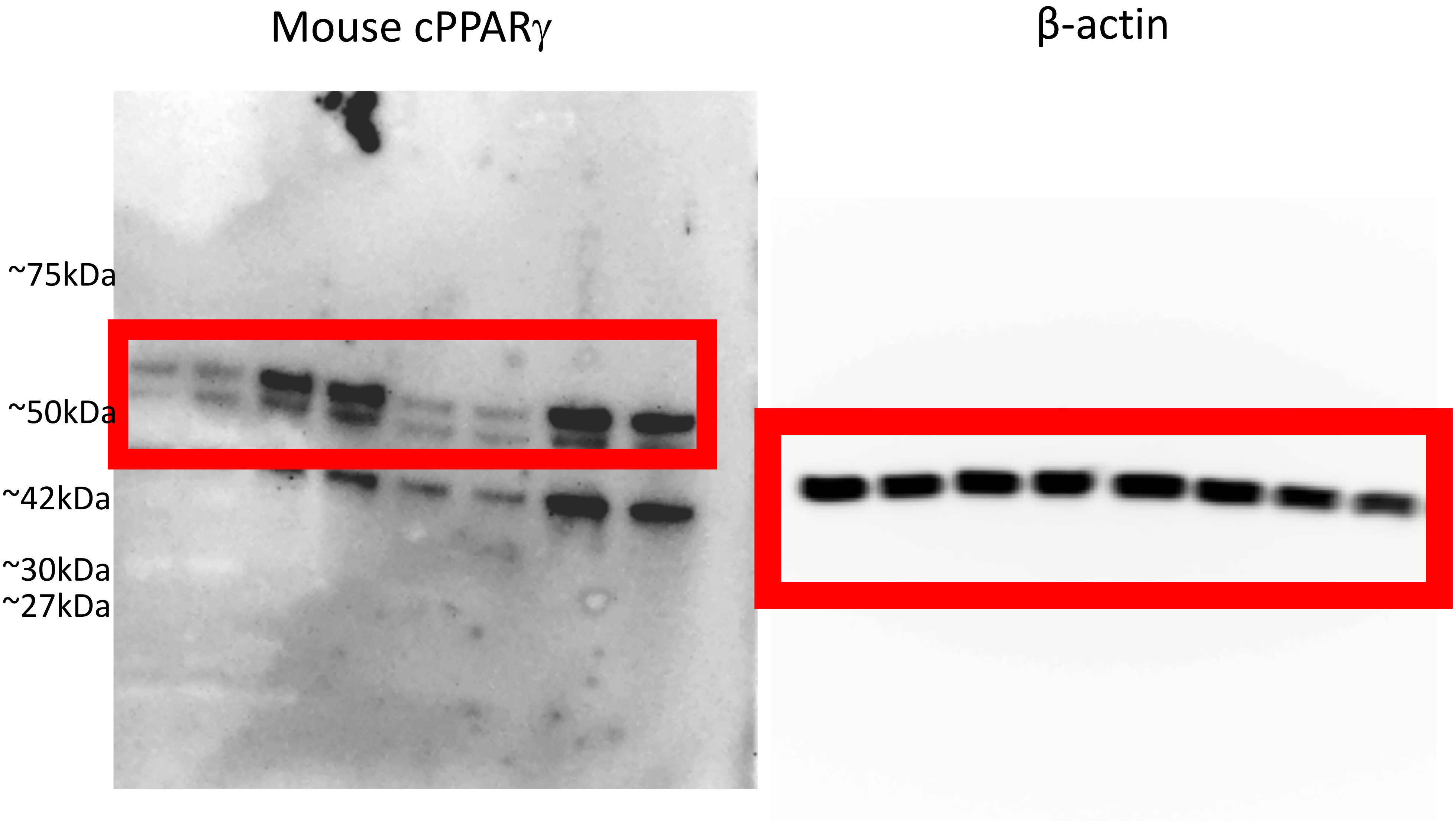

Supplementary Fig. 1A (original whole gel/blot)

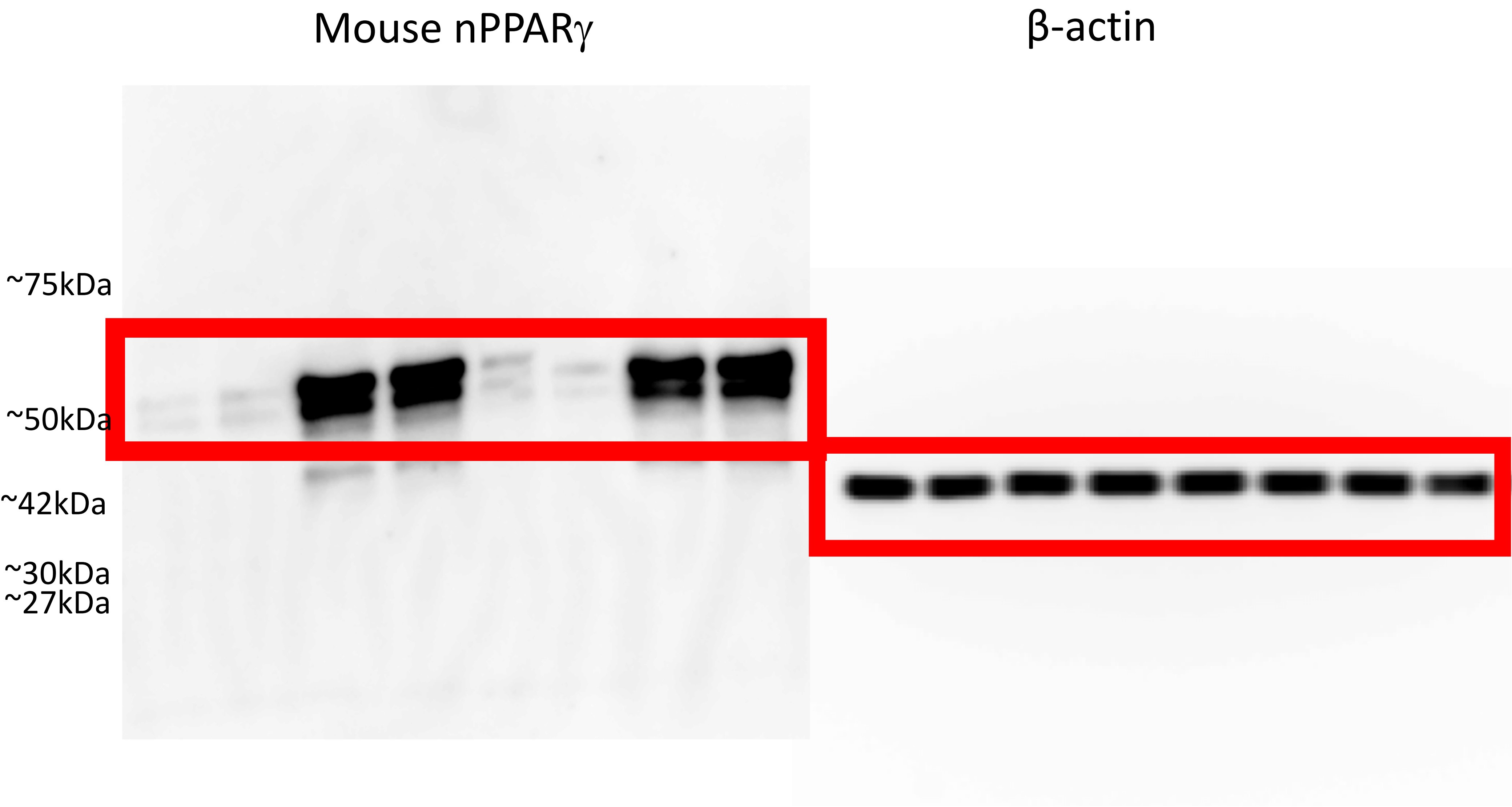

Supplementary Fig. 1E (original whole gel/blot)

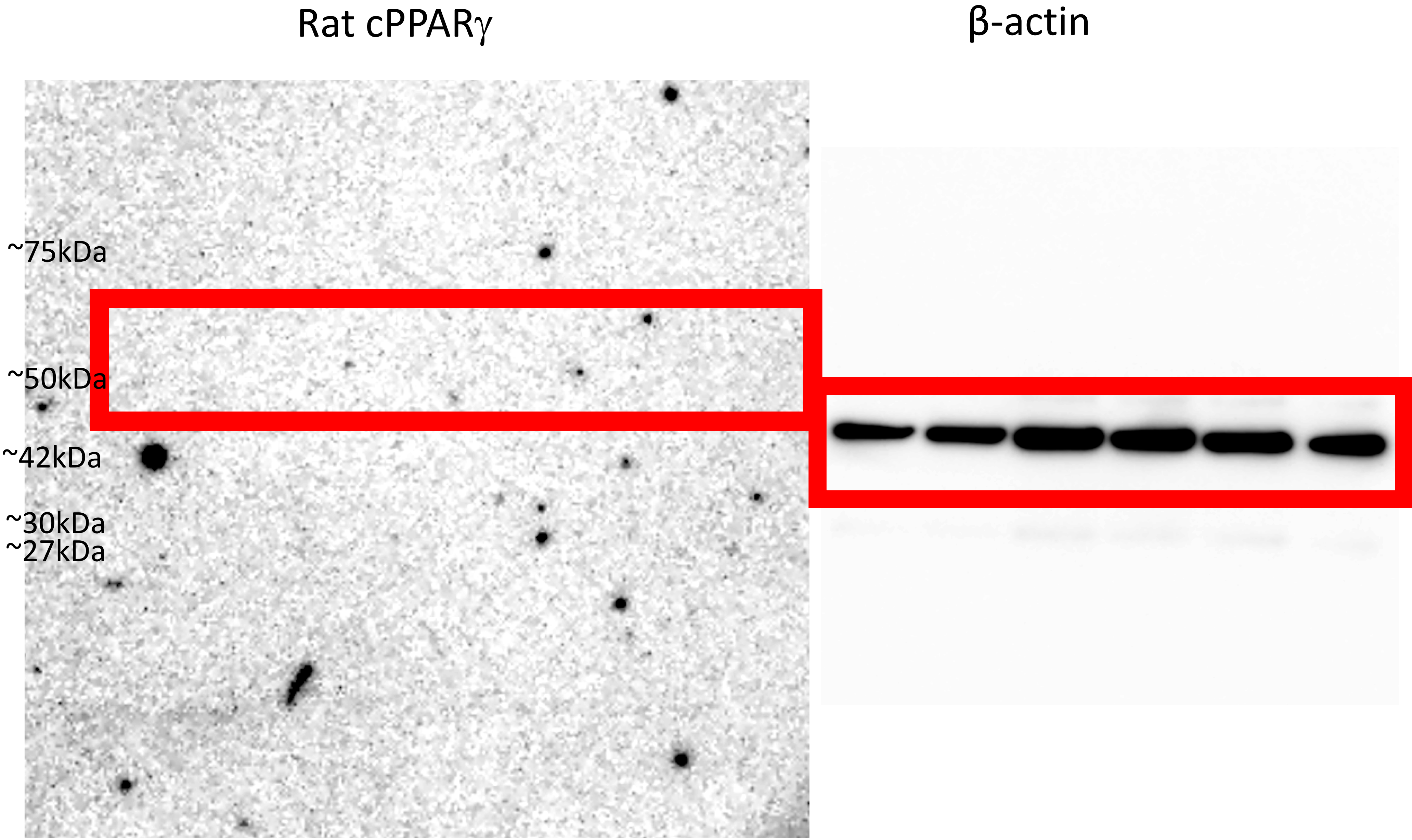

Supplementary Fig. 1E (original whole gel/blot)

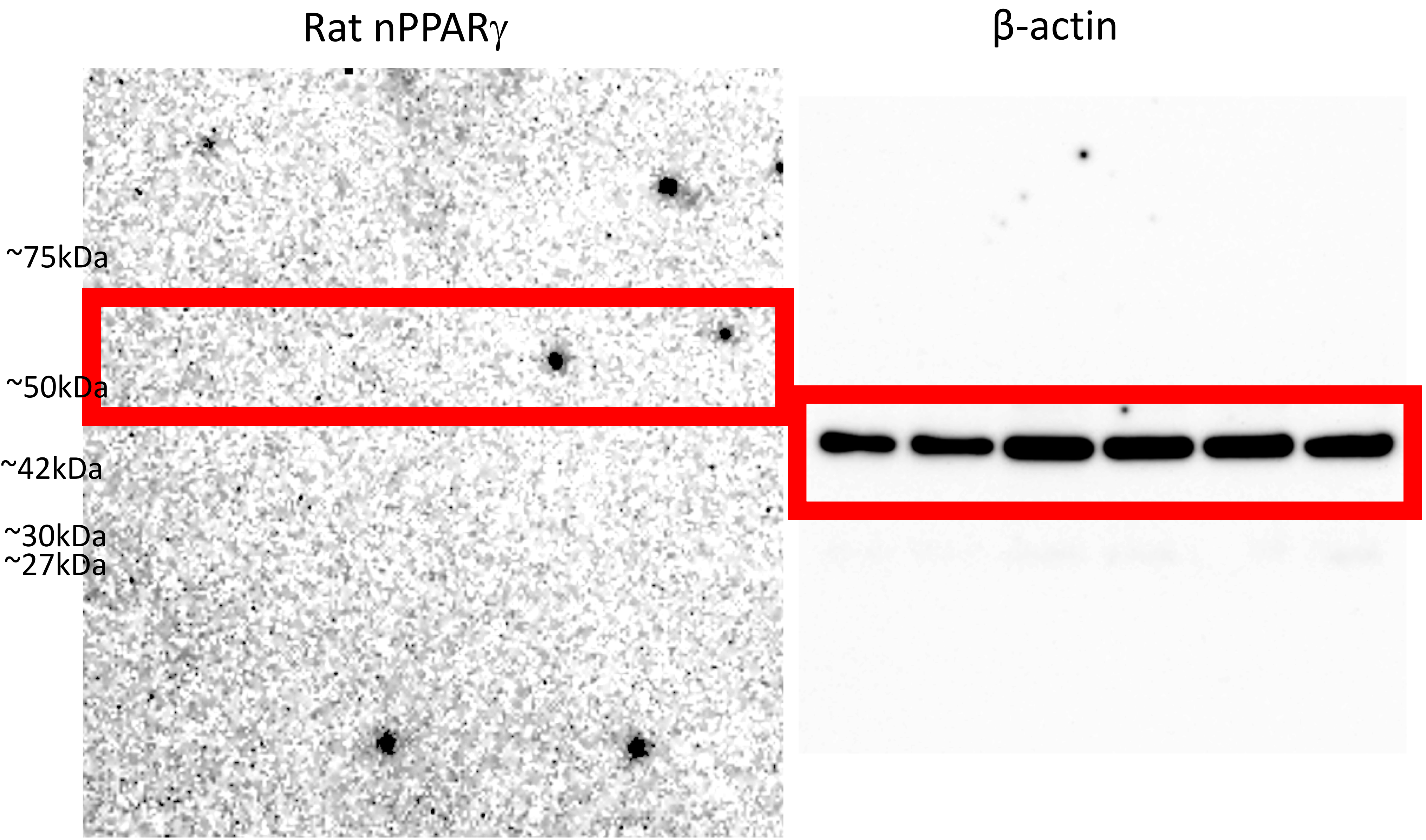

Supplement: Supplementary file 1 — Supplementary Information. [file 41598_2021_4036_MOESM1_ESM.pdf]
